# The Varied Role of Efflux Pumps of the MFS Family in the Interplay of Bacteria with Animal and Plant Cells

**DOI:** 10.3390/microorganisms7090285

**Published:** 2019-08-22

**Authors:** Martina Pasqua, Milena Grossi, Alessandro Zennaro, Giulia Fanelli, Gioacchino Micheli, Frederic Barras, Bianca Colonna, Gianni Prosseda

**Affiliations:** 1 Istituto Pasteur Italia, Dipartimento di Biologia e Biotecnologie “C. Darwin”, Sapienza Università di Roma, Via dei Sardi 70, 00185 Rome, Italy; 2 Istituto di Biologia e Patologia Molecolari, Consiglio Nazionale delle Ricerche (CNR), P.le A. Moro 5, 00185 Roma, Italy; 3 Département de Microbiologie, Institut Pasteur, 75015 Paris, France; 4 Équipe de Recherche Labellisée (ERL) Microbiology, Centre National de la Recherche Scientifique (CNRS), 13009 Marseille, France

**Keywords:** efflux pumps, MFS family, multidrug resistance, bacteria-host interactions, virulence

## Abstract

Efflux pumps represent an important and large group of transporter proteins found in all organisms. The importance of efflux pumps resides in their ability to extrude a wide range of antibiotics, resulting in the emergence of multidrug resistance in many bacteria. Besides antibiotics, multidrug efflux pumps can also extrude a large variety of compounds: Bacterial metabolites, plant-produced compounds, quorum-sensing molecules, and virulence factors. This versatility makes efflux pumps relevant players in interactions not only with other bacteria, but also with plant or animal cells. The multidrug efflux pumps belonging to the major facilitator superfamily (MFS) are widely distributed in microbial genomes and exhibit a large spectrum of substrate specificities. Multidrug MFS efflux pumps are present either as single-component transporters or as tripartite complexes. In this review, we will summarize how the multidrug MFS efflux pumps contribute to the interplay between bacteria and targeted host cells, with emphasis on their role in bacterial virulence, in the colonization of plant and animal host cells and in biofilm formation. We will also address the complexity of these interactions in the light of the underlying regulatory networks required for the effective activation of efflux pump genes.

## 1. Introduction

Efflux pumps (EPs) are found in all living organisms [1,2,3]. In many bacteria EPs extrude a wide range of antibiotics, strongly contributing to the emergence of multidrug resistance (MDR) [4,5,6,7]. As this poses an enormous threat to public health, MDR EPs have been mainly studied in clinically relevant bacteria. Several investigations suggest that the genes encoding MDR EPs cannot be simply considered as the result of recent evolution facilitated by intense antimicrobial therapy, but rather appear to be ancient genes encoding cellular transmembrane devices deeply involved in the physiology and ecology of all organisms [3,8,9,10,11,12,13]. This view is consistent with the fact that MDR EPs can extrude a large variety of compounds besides antibiotics, e.g., bacterial metabolites, heavy metals, plant signaling compounds, organic pollutants, quorum-sensing molecules, and virulence factors. This high versatility makes EPs relevant players in the maintenance of cellular homeostasis, in bacterial interactions with plant and animal hosts, in biofilm formation, and in the adaptation of bacteria to an assorted range of habitat [7,10,11,12,14,15]. Interestingly, the number of MDR EPs is proportional to genome size [9,16], which in turn is known to be dependent on the ecological behavior of bacteria. Free-living organisms generally have large genomes, carrying all genes required for the colonization of different environments [17]. They host a much higher number of MDR systems as compared to endosymbionts or to some pathogens, which have smaller genomes as a consequence of reductive evolution [18,19]. In general, bacterial species from the same genus share most EP genes, although some variability exists due to EP determinants carried by mobile genetic elements.

Based on several criteria, such as sequence similarity, energy source, transport function and substrate specificity, EPs have been traditionally grouped into five large families: The ATP-binding cassette (ABC), the major facilitator (MFS), the resistance-nodulation-cell division (RND), the multidrug/oligosaccharidyl-lipid/polysaccharide (MOP) flippase, and the drug/metabolite transporter (DMT) families [2,4,7]. Two new transporter families, the proteobacterial antimicrobial compound efflux (PACE) family [20] and the p-aminobenzoyl-glutamate transporter (AbgT) family [21], were recently identified. Proteins belonging to the PACE family transport biocides, e.g., acriflavine, while those of the AbgT family are involved in the transport of sulphonamides. ABC transporters directly couple the efflux function to ATP hydrolysis, whereas members of the other EP families are powered by electrochemical gradients across the inner membrane.

The structure of MDR EPs of the MFS family and their specific role in antibiotic resistance has been widely covered by several recent reviews [6,7,13,22,23,24,25,26,27,28]. In this review we focus on other roles of these EPs when bacteria interact with plant, animal and microbial cells. In particular, we address their involvement in bacterial virulence, in the colonization of host cells and in biofilm formation. We also describe the main strategies adopted by bacteria to allow efficient and coordinate expression of MFS EPs in response to environmental stimuli.

## 2. Organization and Evolution of the MFS EPs

In both Gram-negative and Gram-positive bacteria EPs are commonly present as single-component efflux transporters in the inner membrane. In Gram-negative bacteria, they can also form tripartite complexes spanning across both membranes [2,28,29]. Multicomponent EPs have been found among the transporters of the ABC, MFS, and RND families and consist of an inner membrane transporter, a periplasmic adaptor (PA, also known as a membrane fusion protein, MFP), and an outer membrane protein, all of them essential for pump functionality [24,25,28,29].

**Figure 1:**
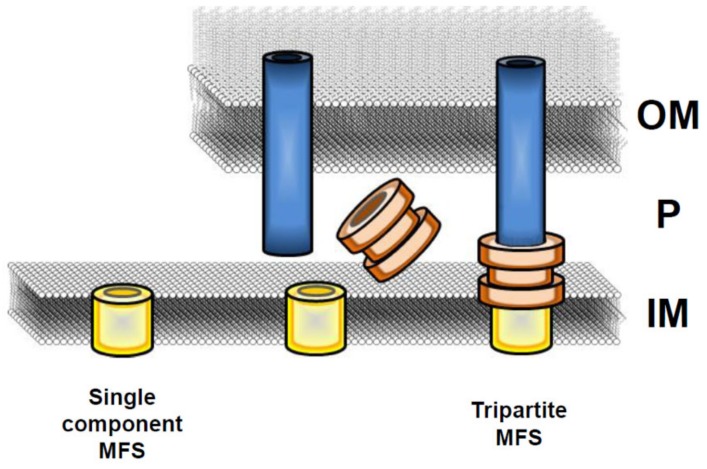
Schematic overview of the architecture of the efflux pumps of the major facilitator superfamily (MFS)**.** Multidrug MFS efflux pumps are typically found as single-component efflux transporters in the inner membrane (IM). In Gram-negative bacteria, MFS transporters can also form tripartite complexes that directly transport substrates from the cytoplasm to the exterior. The inner membrane component does not extend into the periplasm (P) but is thought to interact with a periplasmic adaptor protein within the inner membrane. The adaptor protein forms a sealed channel between the inner and outer membrane (OM) components. The outer membrane channel is constituted usually by the TolC protein. In some cases a TolC-like protein, encoded by the same operon as the inner membrane and periplasmic components, acts as an outer membrane channel.

**Figure 2:**
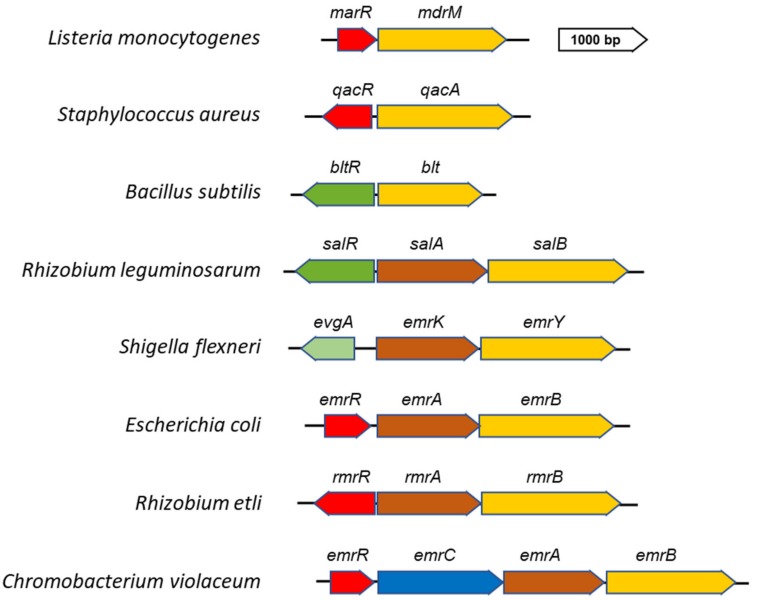
Genetic organization of representative MFS efflux pumps (EP) encoding genes and their local regulators. MFS transporters are encoded by chromosomal or plasmid genes. The genes encoding the inner membrane transporter (yellow) and the periplasmic adaptor protein (brown) are part of a single operon. The outer membrane channel protein (blue) may be encoded by the same operon or by another genetic locus, as in the case of the *tolC* gene. The transcriptional regulation of MFS EP genes is controlled mainly by local regulatory genes encoding repressors (red) or activators (green). In general, local regulators allow the expression of MFS EP operons in response to the substrates they export. Repressors belong to the TetR-, MerR- or MarR- families while activators belong to LysR- or MerT-families. The *evgA* gene (light green) encodes the regulator of a two-component system (EvgA/EvgS) which activates the *emrKY* operon in response to high concentrations of alkaline metals under mild acidic concentrations, as found inside macrophages. Global regulators are not shown in the figure.

The MFS EP family constitutes the largest group of secondary membrane transporters and is present in all phyla, from bacteria to plants and mammals [27,30,31,32]. MFS transporters function mostly in the uptake of sugars, but several MFS proteins are also involved in drug efflux systems, thus contributing to antibiotic resistance both in Gram-negative and Gram-positive bacteria. The MDR MFS transporters are widespread among microbial genomes and generally act as single-component pumps able to transport small solutes across the inner membrane (Figure 1). In Gram-negative bacteria, several MDR MFS pumps are constituted by a tripartite complex [28,29,32] encoded by genes organized in a single operon. In particular, the gene coding for the outer membrane protein is followed by the genes encoding the periplasmic adaptor and the inner membrane transporter. Most often a regulatory gene, either activator or repressor, lies beside and is transcribed independently from the EP encoding genes (Figure 2). The organization of MFS tripartite EPs encoding genes is reversed as compared to RND EPs, where the gene encoding the transporter is always located in a promoter-proximal position followed by the gene coding for the periplasmic protein and then, when present, by the gene encoding an outer membrane component [11]. 

Bioinformatic analyses based on the alignment of conserved motifs suggest that the MFS transporters can be divided into two groups, containing 12 or 14 transmembrane helices or segments (TMS) [27,32]. Despite this variation, the current hypothesis is that both groups have evolved from a simple prototype carrying a hairpin structure with two TMS [32]. This protein may have triplicated into a larger molecule containing six TMS, which in turn may have formed a 12-TMS structure by a successive duplication event. MFS transporters with 14 TMS have two extra, centrally positioned, TMS that are believed to result from intragenic duplication. These transporters can give rise to tripartite pumps which directly transport substrate from the cytoplasm to the outer environment [24,28,29]. The crystal structure of EmrD, a MDR MFS protein, reveals the general architecture of the 12-TMS drug transporters [33]. While eight TMS form an internal cavity, the remaining ones face towards the outside. Most residues in the cavity are hydrophobic, in line with the role in transporting lipophilic molecules, and are conserved in other transporters of the MFS family. According to the classical view, once the drug has entered the cavity, it is transported through a rocker-switch model [33,34]. Recent structural evidence has led to an update, the so-called clamp-and-switch model: A two-step mechanism which better accounts for the structural changes occurring in individual transmembrane helices [27]. In particular, bending of the helices lining the internal pore is assumed to give rise to an occluded state (clamping step). Then the domains would alternatingly rotate, exposing the binding domain to one or the other side of the inner membrane (switching step). 

Exit of substrates through the outer membrane occurs, in most cases, via the TolC protein. TolC is a trimeric outer membrane protein characterized by the presence of an α-barrel projecting across the periplasm and linked to a β-barrel domain embedded in the outer membrane [35]. TolC serves as an external channel not only for the EPs of the MFS family, but also for EPs of the RND and ABC families, thus showing a remarkable substrate versatility. TolC-like proteins are often encoded by the MFS operon [24,36,37] (Figure 2) indicating that in a wide range of bacteria, TolC-type features are a structural requirement for the assembly of a tripartite MFS EP [29,35]. The architecture of tripartite efflux pumps provides a continuous seal so that exported compounds can bypass the periplasm (Figure 1). In the case of RND tripartite EPs, the seal is ensured by a close fit between the periplasmic domains of TolC and of the inner membrane component, which is further stabilized by the adaptor component of the pump [11,25,29]. Structural studies of the MFS EmrAB-TolC EP of *Aquifex aeolicus* suggest that the inner membrane transporter does not contain significant periplasmatic domains and the seal is likely the result of the interaction between the adaptor protein and the periplasmatic domain of TolC [23].

## 3. Transcriptional Strategies for the Regulation of MFS EPs 

The dynamic adaptation of bacteria to the highly diverse habitats relies on their capability to quickly sense environmental variations and respond in a way which frequently implies severe changes in the transcriptional profile of the cell. As EPs are able to extrude a wide range of structurally unrelated chemicals, it is reasonable to assume that their altered expression may lead to an unbalanced efflux of metabolites or other signaling molecules and adversely affect cell physiology. Not surprisingly, the expression of EPs is usually well-tuned and, under physiological conditions, occurs only at a low level [13,38]. Remarkably, different bacterial species share common EP regulatory circuits: Expression patterns found in aerobic bacteria are also present in microaerobic and anaerobic species [38,39].

The regulation of MFS EP synthesis most often relies on a transcriptional repressor belonging to the TetR-, MarR- or MerR-type [38]. Activation occurs after an effector binds to the repressor protein, making it unable to exert its function. A well-known example of this regulation is the induction of *E. coli emrAB* by inactivation of the EmrR repressor [40]. The *emrAB* operon encodes the EmrB transporter and the EmrA periplasmic adaptor of a MDR tripartite EP (Figure 2). As an outer membrane component, the EmrAB EP uses the TolC protein. The *emrR* gene is located immediately upstream the *emrAB* genes and is independently transcribed. EmrR binds directly to the promoter region of *emrAB* and inhibits its transcription under non-inducing conditions [41]. Toxic chemicals, which act as EmrAB substrates (e.g., carbonylcyanide-3-Chlorophenylhydrazone, 2,4–dinitrophenol (CCCP) or ethidium bromide), bind to EmrR inducing conformational changes that weaken its interaction with the promoter, thus relieving *emrAB* repression. Additionally, in the human pathogen *Vibrio cholerae* VceCAB, an MDR EP sharing homology with EmrAB, is negatively regulated by a TetR-like repressor VceR. VceCAB confers resistance to several antibiotics, CCCP and deoxycholate and is encoded by a large operon also including the outer membrane component VceC [42]. VceC has a high degree of structural similarity with TolC although a wide divergence exists at the primary sequence level [43]. The mechanism leading to the derepression of the *vceCAB* operon has been studied in detail using CCCP as pump substrate. In particular, it has been shown that VceR is able to bind CCCP but that its affinity for this substrate decreases in the presence of DNA, suggesting that the equilibrium between free VceR and CCCP-bound VceR is critical for the synthesis of the pump [44]. 

A further example of negative control of MFS EP genes is represented by the *farAB* operon, which encodes an EP system that mediates resistance of *Neisseria gonorrhoeae* to antimicrobial long-chain fatty acids [45]. The expression of *farAB* operon is negatively controlled by the FarR repressor, belonging to the MarR family, which binds to three sites within the promoter region [46]. Repression by FarR is enhanced by binding of the nucleoid protein IHF to the *farAB* promoter, inducing DNA curvature. This conformational change stabilizes the FarR-DNA complex and results in strong repression of *farAB* [47], stressing the relevance of DNA bending in the regulation of gene expression in bacteria [48,49]. Expression of *farAB* is also controlled by MtrR, a regulator that normally represses *mtrCDE*, which encodes another EP involved in resistance to long-chain fatty acids. MtrR plays an indirect role as it does not bind directly to the *farAB* promoter, but rather represses the transcription of *farR* and thus alleviates the FarR-mediated repression of *farAB* [46]. Other EPs sharing homologies with *E. coli* EmrAB exist in several bacteria interacting with plant and animal cells as will be discussed in detail in the following sections. Here, we just stress that also in these cases, repression by an EmrR-like protein is relieved in response to stimuli from the host.

In addition to EmrAB-like pumps, other examples of MFS EP genes submitted to negative control are worth mentioning. In *Listeria monocytogenes*, the expression of the MdrT pump is under the negative control of the TetR-like repressor BtrA [50]. BtrA loses the ability to bind to and repress the *mdrT* promoter in the presence of cholic acid, thus facilitating bacterial survival in host bile-rich environments. BtrA is also responsible for the cholic acid-dependent induction of another MFS efflux pump, MdrM, which is negatively controlled by the local repressor MarR [50,51]. Another case of repressor released from the promoter following binding of specific ligands is QacR, the regulator of the multidrug transporter QacA. QacA has been among the first bacterial MDR transporters identified [52] (Figure 2). It is encoded by plasmids as pSK1, frequently associated with *Staphylococcus aureus* and other relevant human pathogens [38]. The cognate encoding regulator gene *qacR* is divergently transcribed from *qacA* and binds to the regulatory region upstream *qacA*, thus inhibiting its transcription [38]. Cationic lipophilic compounds, as well as several synthetic antimicrobial substances, bind to QacR resulting in its dissociation from the promoter, which in turn enables expression of *qacA*. Interestingly, QacA is also activated by the plant alkaloid berberine. These observations suggest that berberine may be a natural substrate of QacA and that the QacA-QacR system, originally providing resistance to plant and other naturally derived antimicrobial compounds, has been successively recruited by bacterial pathogens to survive in the presence of antimicrobial agents used in clinical therapies [38]. An interesting regulatory pathway is represented by Tet38, another MDR EP of *S. aureus* [53,54]. Transcription of *tet38* is repressed by a TetR-like regulator, TetR21. Tet38 substrates, such as tetracycline or palmitoleic acid, disrupt the TetR21-*tet38* promoter complex, allowing expression of the *tet38* containing operon [54]. The *tet38* gene is also under the negative control of the global regulator MgrA [55]. MgrA is able to bind to the *tetR21* promoter but not to the *tet38* promoter, suggesting that it indirectly contributes to the repression of *tet38* by increasing the transcription of *tetR21.* Another *S. aureus* MDR EP, NorB, is under the direct control of MgrA [55]. In its phosphorylated form MgrA binds as a dimer to the *norB* promoter, repressing its transcription. The *norB* gene is highly expressed in the host environment, especially in abscesses and is important for survival in such locations [56]. The relative expression level of *norB* is also found up-regulated during *S. aureus* biofilm growth [57]. Under the mildly acidic and low-oxygen environment found in abscesses and possibly within the core of biofilms, MgrA undergoes post-transcriptional modifications, which result in alleviation of its repressive activity on *norB* [55]. In contrast to NorB and Tet38, NorD, a further MDR MFS EP of *S. aureus*, is not submitted to the control of MgrA. The *norD* gene is repressed by Fur and is induced in iron-restricted environments, a condition which bacteria frequently encounter within mammalian hosts [58].

The expression of MFS pumps can also be activated by transcriptional regulators which, in response to specific inducers, facilitate the binding of RNA polymerase to the corresponding promoters. This is exemplified by Bmr, an MFS multidrug transporter *of Bacillus subtilis*. It is positively regulated by BmrR, a MerR-like regulator encoded in the same locus [59]. BmrR is activated by a large range of structurally diverse hydrophobic molecules, including toxic compounds transported by Bmr [38]. Binding of BmrR to the *bmr* promoter induces a conformation compatible with intense transcription. A complex mechanism based on localized base-pair breaking, base sliding and realignment allows BmrR to upregulate transcription by reconfiguring the 19-base-pair spacer between the -35 and -10 promoter elements for highly productive interaction with RNA polymerase. Besides BmrR, also another MerR-like regulator, the global regulator Mta, activates the *bmr* gene by binding the same promoter site as BmrR. A similar circuit, requiring both local and global positive regulators, activates Blt, an MDR EP sharing 50% aminoacid identity with Bmr [59]. In contrast to *bmrR* the gene encoding the local regulator *bltR* is divergently transcribed from the *blt* gene and is not activated by Blt substrates (Figure 2). Blt has been shown to be involved in the efflux of polyamines [60], small molecules emerging as relevant players in bacteria-host interactions [61,62]. In this case, the Mta global regulator is also required for the full expression of *blt* gene [38]. Another operon submitted to positive control is *salAB* in *Rhizobium leguminosarum* [63]. It is activated by SalR, a LysR-like regulator, in response to phenolic compounds released by the plant (Figure 2).

EPs can also be controlled by two-component signal transduction systems (TCS). These systems typically respond to environmental conditions by means of an inner membrane histidine kinase that senses and transduces different signals phosphorylating a response regulator. A global analysis of the role of TCS in the regulation of *E. coli* EPs [64] has evidenced that TCS systems like BaeSR, CpxAR, and EvgAS control several pumps in response to different stimuli. Among MFS efflux pumps, EmrKY is regulated by EvgAS in mildly acidic media and under high concentrations of alkaline metals [65,66]. The EvgAS system is encoded by an operon divergently transcribed from the *emrKY* genes (Figure 2). Recently, it has been observed [66] that the same stimuli (K^+^ and acidic pH) acting on the EvgAS system also control *ermKY* expression in *Shigella flexneri*, a human life-threating pathogen [67,68]. In particular, the analysis of *emrKY* expression during the intracellular life of *Shigella* has shown that the same regulation also occurs inside macrophages, where, following *Shigella* infection, the pH decreases favoring the activation of *emrKY* transcription by the EvgAS system [66]. 

## 4. Role of MFS EPs in Interactions Between Bacteria and Plant Cells

Plants produce a large array of secondary metabolites, such as phytoalexins and alkaloids, which protect them from pathogens [69]. On the other hand, bacterial plant pathogens have evolved systems to counteract this chemical barrier. Several studies have shown that, among these systems, MDR tripartite EPs are key elements in conferring resistance to plant toxic compounds, thus facilitating the colonization of the host by bacteria [3,7,15]. In addition, EPs are also involved in interactions between plants and symbiotic bacteria such as rhizobia, well known for their capacity to form nitrogen-fixing nodules on the roots of legumes. Within the RND EP family several cases have been described illustrating the involvement of these pumps in resistance to toxic compounds, nodulation and interspecies signal trafficking [11,12]. Some MFS EPs are relevant to plant-cell interactions in both pathogenic and symbiotic bacteria (Table 1). Two MFS EPs, Emr1AB and Emr2AB, analogous to the *E. coli* EmrAB EP, have been shown to be involved in the virulence of *Erwinia chrysanthemi*, the causative agents of soft root disease [70]. Virulence of *E. chrysanthemi* is a complex process that depends on several factors, including plant cell wall degrading enzymes causing maceration and necrosis of plant tissues [71]. Some interesting differences have been outlined comparing the virulence of *emr1AB* or *erm2AB* mutants in infection assays with different host plants. Indeed, while infection with *E. chrysanthemi emr2AB* mutants gives rise to a reduced necrotic area in African violet and in chicory leaves, *emr1AB* mutants exhibit reduced virulence only in the African violet host. The fact that *emr2AB* mutants do not grow in chicory leaves suggests that the lack of Emr2AB drastically affects the ability of *E. chrysanthemi* to initiate plant colonization. On the other hand, mutants defective in *emr1AB* are extremely sensitive to potato extracts and their ability to infect potato tubers is severely reduced [70]. The toxic compound present in chicory and in potato tuber extracts has not been identified. Besides sharing the ability to confer resistance to a large panel of antibiotics, Emr1AB and Emr2AB also provide resistance to oleic acid and, respectively, to all phytoalexins. Taken together, these data suggest that the function of these EPs may be related to the transport of specific toxic compounds found only in a given plant host. Moreover, Ravirala et al. [72] have demonstrated that the expression of *emrA* in *E. chrysantemi* 3937 is increased three-fold in the presence of salicylic acid and up to five-fold in response to a combination of phenolic acids (including salicylic acid, benzoic acid and trans-cinnamic acid), thus confirming that the exploitation of plant defence signaling by bacteria to activate EPs may have evolved to increase resistance to toxic molecules and thus ease survival in a hostile environment.

As for the involvement of the MFS EPs in symbiotic interactions between bacteria and plants, specific systems have been characterized in *Rhizobium etli*, *Sinorhizobium melitoti*, and *R. leguminosarum* [63,73,74,75,76]. A flavonoid inducible MFS efflux pump, RmrAB, has been described in *R. etli*, a mutualistic symbiont of *Phaseolus vulgaris*. The *rmrA* and *rmrB* genes are divergently transcribed from a gene encoding a regulator of the TetR family [74] (Figure 2). Mutants defective in *rmrA* or *rmrB* form fewer nodules on bean roots and have an increased sensitivity to toxic compounds as naringenin, phytoalexins and salicylic acid. Interestingly, the ability to grow in the presence of a high concentration of naringenin is restored in the presence of a plasmid encoding the *E. coli* EmrAB pump [74]. Besides underlining the functional similarity between the *E. coli emrAB* genes and the *rmrAB* genes of *R. elti*, this study reveals how MFS EPs are involved in nodule formation and in resistance to plant flavonoids. A systematic study of efflux systems in rhizobia has been carried out in *S. melitoti*, a symbiont of alfalfa, to better understand the effect of the loss of MDR EP genes on the resistance to antimicrobials and on symbiosis [76]. *S. melitoti* interacts with legume roots inducing the formation of nodules within which the bacterium fixes nitrogen to ammonia that becomes available to the plant. By bioinformatic analysis several MDR tripartite EP systems have been identified [76], including three belonging to the MFS family. Among them there is an EmrAB system which shares homologies with the *E. coli* EmrAB EP. The expression of the *emrAB* gene of *S. melitoti* is under the control of a TetR-like EmrR repressor [73,77] and occurs in response to stimuli from the plant. Using an *emrA*::*gusA* fusion it has been shown that *emrAB* genes are induced by several flavonoid compounds as luteolin, quercetin, galangin, naringenin and apigenin produced by the plant in response to bacterial colonization [77]. In particular, it has been shown that in vitro luteolin interferes with the binding of the EmrR repressor to the *emrAB* regulatory region, accounting for the higher expression of the *emrAB* operon in response to flavonoids. Deletion of the *emrAB* operon does not affect neither the *S. melitoti* sensitivity to several toxic compounds nor the symbiotic properties. While these data seem to rule out an involvement of this EP in the symbiotic process, expression profiling analysis shows that the *emrAB* operon and the *ermR* regulatory gene are also inducible by heat shock and low pH, indicating a possible role for this EP in the response to stress [75]. Moreover, the loss of EmrR strongly decreases the ability to form nodules and the competitiveness against the wild-type in co-inoculation experiments with Medicago sativa as host [73]. This suggests that EmrR also regulates other genes of *S. melitoti* and that it is an important player for the successful interaction of the bacterium with the plant.

A key molecule in the response of plants to microbial colonization is salicylic acid, a phenolic hormone with multiple roles in plant metabolism and physiology [78]. Amongst *R. leguminosarum bv viciae* 3841 genes upregulated in response to salicylic acid, two operons, *salAB* and *rmrAB*, encode MFS EPs [63]. The *rmrAB* genes are located on a plasmid and share high homology with the corresponding system of *R. etli* CFN42 [74]. The *salAB* genes represent a novel system, which is organized in an operon divergently transcribed from the *salR* regulatory gene (Figure 2). SalR is specifically activated by salicylic acid and to a lesser extent by acetylsalicylic acid [63]. Mutagenesis analyses reveal that deletion of *salA*, but not of *rmrA*, significantly increases the sensitivity to salicylic acid [63]. This is in contrast with the observed capacity [74] of the RmrAB pump to confer resistance to salicylic acid in *R. etli*. Another relevant difference, as compared to *R. etli* is found at the functional level: in pea plants, it has been observed that mutants lacking *salA* or *rmrA* do not exhibit alterations in the nodulation capacity or in the rate of nitrogen fixation [63]. While this is expected for *salA*, as it is expressed at low levels in nodule bacteria, it is particularly surprising for RmrAB since its genes are highly upregulated (more than 20-fold) during bacteroid development. This opens the possibility of a novel, yet undefined role of the RmrAB pump in plant-bacterium interactions.

## 5. Role of the MFS EPs in the Virulence of Bacterial Pathogens in Animal Hosts

Several studies indicate that MDR MFS EPs actively contribute to the virulence of bacterial pathogens. These EPs can be required for colonization and dissemination during host infection, being involved in the resistance to defence compounds produced by the host (Table 1). In some cases they can be directly implicated in the invasion process. Examples of MFS EPs that mediate resistance to host-derived antimicrobials, favoring pathogen dissemination in the host, are found in *N. gonorrhoeae* and *L. monocytogenes*.

It has been demonstrated that the FarAB EP mediates resistance of *N. gonorrhoeae* to long-chained fatty acids (FA) such as oleic, linoleic and palmitic acids [45]. *N. gonorrhoeae* is a strict human pathogen causing gonorrhea, a sexually transmitted disease. Although the gonococcus is most often responsible for urogenital infections it can infect other mucosal surfaces, such as the rectum or the oropharynx mucosae. Several host antibacterial mechanisms operate at different infection sites. The major actor conferring resistance to antimicrobial compounds present on mucosal sites is the RND pump MtrCDE, which is involved in the export of several host-derived hydrophobic agents [94]. A study by Lee and Shafer [45] identified FarAB as a second efflux system involved in gonococcal resistance to a specific subset of long-chained FAs. Indeed, the authors were able to demonstrate that *farB* insertional mutants of clinical isolates have acquired high susceptibility to those FAs. The *far* (for “FA resistance”) system is composed of the FarA adaptor protein and of the FarB cytoplasmic membrane transporter. Both display sequence similarity with the *E. coli* EmrAB. This EP requires the MtrE protein as outer membrane channel to export antibacterial FAs from inside the cell. As discussed, the expression of *farAB* is negatively regulated by FarR, which, in turn, is repressed by MtrR, the repressor of the *mtrCDE* EP [46,95]. The opposite activity of MtrR on the *mtr* and *far* operons likely prevents abnormal expression of EPs, which has been demonstrated to be harmful to gonococci [96]. At the same time it provides gonococci with an alternative way to counter host-innate defences, as in the case of resistance to the antibacterial effects of FAs present in faecal lipids observed in a subset of rectal isolates [97]. 

A study assessing the transcriptional response of *L. monocytogenes* to mammalian bile has identified that two MDR MFS EPs, MdrM and MdrT, are strongly induced by the bile component cholic acid [50]. Mammalian bile displays a potent anti-microbial activity. Bacterial pathogens of the gastrointestinal tract and the hepatobiliary system must be able to survive in these bile-rich environments in order to colonize host cells and disseminate during infection [98]. *L*. *monocytogenes* is a foodborne pathogen, which, initially resides within the intestine and then crosses the intestinal barrier, rapidly disseminating to liver and spleen, the primary target organs. The bacterium can also persist extracellularly within the gallbladder lumen, where bile is highly concentrated. Several genes contribute to the survival of *L. monocytogenes* in bile-rich environments [99]. The *mdrM* and *mdrT* efflux pump encoding genes are among them and are strongly induced by cholic acid. However, only one of the two pumps, MdrT, is able to extrude this compound and this results in the capacity of *L. monocytogenes* to survive in a bile-rich environment. The deletion of *mdrT* attenuates growth in the presence of cholic acid in vitro and causes a 100-fold reduction of mouse gallbladder colonization in vivo. The MdrT EP is thus regarded as an important virulence factor of *L. monocytogenes* [50]. As far as MdrM is concerned, it has been suggested that it can synergize with MdrT in *L*. *monocytogenes* mouse liver colonization although the exogenous substrates of this EP have not yet been identified [50].

The role of the MFS MdrM and MdrT EPs is not limited to bile resistance. Indeed, they are known for their involvement in the pathogenesis of *L. monocytogenes* and for their ability to modulate the cytosolic surveillance pathway of innate immunity, favoring bacterial intercellular spread and tissue invasion. In particular, ectopic expression of both MdrM and MdrT leads to the massive activation of IFN-β in infected mouse macrophages, while deletion of the *mdrM* gene reduces the secretion of IFN-β, suggesting that MdrM actively controls the capacity of cytosolic bacteria to induce IFN-β expression [51]. Taking advantage of a *L. monocytogenes* strain overexpressing *mdrT* it was possible to identify the molecule secreted by MdrT as cyclic-di-AMP. It has been suggested that under physiological conditions MdrM stimulates IFN-β production by transporting this natural, non-toxic substrate [79]. Subsequently, other MdrM-homologs were identified. Together with MdrM they are responsible for most of the IFN-β induction during *L. monocytogenes* infection of mouse macrophages [100]. These molecules, collectively referred to as MTAC transporters (for MdrM, MdrT, MdrA, and MdrC), are highly induced during bacterial infection. Although they are not necessary for bacterial growth in macrophage cells their lack impairs *L. monocytogenes* virulence in mice [100]. In these studies c-di-AMP released by MTAC transporters has clearly shown its capacity to induce a controlled activation of cytosol sensing pathways, which appears to be beneficial to the *L. monocytogenes* infectious process in vivo. More recent findings have shed light on how secreted cyclic-di-AMP is pivotal for *L. monocytogenes* virulence. It has been demonstrated that the primary sensor for this cyclic dinucleotide is the oxidoreductase RECON (a reductase controlling NF-κB) [101] which upon binding of c-di-AMP, releases the negative control on NF-κB activation downstream TLR (Toll-like receptor) engagement, eventually leading to nitric oxide production. High nitric oxide improves *L. monocytogenes* intercellular spread, the most consistent virulence feature of epidemic strains [102]. MTAC transporters also play a role in the stress response of the *L. monocytogenes* cell wall. It has been suggested that MTAC transporters are involved in enhancing peptidoglycan synthesis under stimuli that produce cell wall stress, such as vancomycin, and that a link between this function and c-di-AMP secretion may exist [100]. Moreover, induction of MTAC-dependent IFN-β in infected macrophages was found to be triggered by *L. monocytogenes* mutants defective in lipoteichoic acid (LTA) synthesis. Under normal growth conditions LTA synthesis itself has been demonstrated to require MDR transporters, possibly via c-di-AMP efflux [103]. 

Another example of an efflux system involved in physiological functions, such as lipid metabolism, and, at the same time crucial for the virulence phenotype, has been described in mycobacteria. It consists of the lipoprotein LprG and the MDR MFS efflux pump P55. LprG and P55 are encoded by the *rv1411c-rv1410c* operon, which is conserved across several non-pathogenic and pathogenic species of mycobacteria, including *Mycobacterium bovis* and *Mycobacterium tuberculosis* [104]. Several studies have demonstrated that this operon is essential for the virulence of various mycobacterium species in mice, as deletion of *rv1411c-rv1410c* reduces bacterial replication in macrophages and survival in mouse models [81,82,105]. In *M. tuberculosis* the LprG-P55 system was found to translocate triacylglycerides (TAG) from the cytoplasm to the outer membrane through a mechanism where P55 transports TAG across the inner membrane and passes it to LprG, which then transfers it to the outer membrane [106]. In the host lipids represent the major source of carbon for *M. tuberculosis* and these conditions favor the production of TAG, which serves as a reliable long-term energy source. It has been proposed that the LprG-P55 transport system critically contributes to preventing abnormal intracellular TAG accumulation during certain growth conditions within the host, protecting the bacteria from toxic byproducts of cholesterol by discarding them via TAG from the cytoplasm [106]. The role of the P55 EP in the pathogenesis of *Mycobacterium* is further stressed by high throughput screening studies indicating that transposon mutants of the *rv1410c* gene are significantly attenuated in mouse models [107] and in macrophages [108]. Accordingly, in *M. bovis* P55 has been found to be involved in cholesterol uptake and required for optimal growth on cholesterol, one of the major sources of carbon in vivo [109]. In mycobacteria the functions of MFS transporters are mostly linked to intrinsic and acquired drug resistance which is responsible for the treatment failures of tuberculosis [110]. However, upregulation of MFS transporters may not necessarily depend on drug stress, and drug tolerance may be a side effect of their physiological role, including virulence. Using a model consisting of Zebrafish larvae infected with *Mycobacterium marinum*, it has been demonstrated that growing mycobacteria develop multidrug tolerance soon after they infect macrophages, independently from drug exposure. Drug tolerance is maintained for hours after macrophage killing and depends on the activity of EPs [83]. In *M. tuberculosis* the Tap efflux pump is encoded by *rv1258c,* one of the genes transcriptionally induced upon macrophage infection [84,85]. Deletion of *rv1258c* results in reduced intracellular growth and hypersusceptibility to rifampicin, indicating the specificity of the Tap EP in mediating macrophage-induced tolerance [83]. Subsequently, in *M. tuberculosis* it has been reported that *rv1258c* is among the genes whose expression is induced in response to the lysosomal soluble fraction (SF) prepared from activated macrophages and that its deletion determines higher susceptibility to killing by lysosomal SF, as well as attenuated survival in macrophages [111]. Tap represents an example of EP induced upon host infection to counteract a hostile cell environment and promote bacterial growth, which is implicated in mediating drug tolerance.

The involvement of the MDR MFS EPs during infections by bacterial pathogens is further highlighted by Tet38, a *Staphylococcus aureus* EP which contributes to the bacterial internalization and survival within host cells [53,54,80]. Tet38 is a chromosomally encoded EP that is responsible for the extrusion of several substrates, including tetracycline, fosfomycin, and unsaturated fatty acids. *S. aureus* is a versatile bacterium able to cause acute and chronic infections in humans and animals due to its large arsenal of virulence factors and its ability to acquire multiple drug resistance. The persistence of *S. aureus* may be due in part to its capability to survive in and adapt to the host intracellular environment, enabling escape from the effects of antibiotics and of the host immune response. Although *S. aureus* is usually not an intracellular pathogen it can invade epithelial and endothelial cells and survive within them. The involvement of Tet38 in the uptake of *S. aureus* by host cells has been clearly demonstrated by observing the severe reduction in the recovery of *S. aureus tet38* mutants after invasion of human epithelial cells [54]. Tet38 interacts with the membrane-associated host cell receptor CD36. Once inside the host cell Tet38 contributes to the escape of internalized *S. aureus* cells from phagolysosomes [80]. Indeed, Truong et al. [80] have shown that while intracellular *tet38 S. aureus* mutants remain mainly associated with the phagolysosome with a progressive decrease in their number due to their failure to replicate, the wild-type strains are present in a greater number of cells not associated with the phagolysosome. In addition to the *tet38* gene, also the *norB* and *norD* genes, encoding two MFS MDR transporters, are strongly upregulated during *S. aureus* infections in a mouse subcutaneous abscess model [53]. Although their specific role and the potential natural substrates within abscesses have not yet been identified, *norB* or *norD* mutants display significant fitness defects in competition assays as compared to the wild-type [56,58]. In particular, in *S. aureus norD* mutants the selective fitness impairment is greater than in *norB* mutants. These data indicate that NorB and NorD EPs not only contribute to increasing the antibiotic resistance, but also facilitate bacterial survival within staphylococcal induced abscesses.

Very recently the MFS EmrKY EP has been demonstrated to facilitate survival of *Shigella* within a macrophage environment [66]. The infectious process of *Shigella*, a human intracellular pathogen, is characterized by the ability to invade macrophages, where it multiplies and induces cell death, before being released and then internalized by the epithelial cells of the colonic mucosa [67,68]. Despite a high genome homology with the commensal *E. coli*, *Shigella* has undergone extensive gene decay [19,112], resulting in the lack of 6 out of 20 MDR EP systems described in *E. coli* [66]. Analyses of the transcription of EP genes in response to the intracellular environment *Shigella* encounters during the infectious process have shown that the EmrKY EP is selectively induced in macrophages but not in epithelial cells [66]. It has been proposed that this may depend on the different cytosolic pH conditions occurring in the two cell types. In particular, the acidic conditions met in macrophages induce *emrKY* via the EvgA/EvgS two-component system. Competition experiments with *Shigella* strains containing or lacking the *emrKY* operon reveal that EmrKY helps *Shigella* to better survive inside macrophages [66]. It has been reported that *E. coli emrKY* mutants, besides having a reduced ability to form biofilms, exhibit hypersensitivity to several drugs, such as mitomycin C, a DNA alkylating agent [113]. On this basis, it has been suggested that EmrKY might contribute to the survival of *Shigella* within macrophages by safeguarding cells from DNA damaging compounds.

## 6. Role of the MFS EPs in Bacterial Communication and Biofilm Formation

Communication between bacteria and the external environment, as well as among bacteria within the same ecological niche, is pivotal for adaptation to new habitats. It allows survival under stressful conditions and, in the case of pathogenic bacteria, is usually highly relevant to the infectious process. A sophisticated intercellular communication mechanism is represented by quorum sensing. Under high cell density signal molecules accumulate and bind to specific transcriptional activators triggering a range of processes, including virulence, biofilm formation, and drug resistance. An interesting case of MFS EP involved in quorum sensing is the MDR EmrCAB EP of *Chromobacterium violaceum*, a Gram-negative opportunistic human pathogen usually found in water and soil. EmrCAB is encoded by a large operon similar to EmRAB but also including the gene for a TolC-like outer membrane component [37] (Figure 2). It is regulated by a MarR-like repressor, EmrR. To control the production of the microbicidal purple pigment violacein this microorganism exploits a quorum-sensing system consisting of a CviI/CviR circuit which produces and receives specific acylated homoserine lactone (AHL) autoinducers [114]. Deletion of the *emrR* gene induces, in addition to antibiotic resistance, a decreased production of violacein. The combined deletion of *emrCAB* and *emrR* restores violacein production [37], confirming that EmrR controls the synthesis of the pigment through repression of *emrCAB*. It has been proposed that a high level of EmrCAB alters the quorum-sensing signaling by exporting long-chain AHL molecules synthetized by CviI and required to activate violacein biosynthesis. An EP similar to EmrCAB, EmRCABsm, has also been characterized in *Stenotrophomonas maltophilia*, a Gram-negative opportunistic pathogen associated to cystic fibrosis and highly resistant to a broad spectrum of antibiotics [36,115,116]. In line with previous observations on homologs of EmrCABs in other microorganisms, this EP is negatively regulated by EmrRsm and favors the extrusion of highly hydrophobic substrates. Considering the ubiquitous nature of *S. maltophilia*, this EP could have a physiological role in allowing the bacteria to survive in their ecological niche or to export signaling molecules [36] (Table 1). 

Efflux pumps are involved in biofilm formation since, in most cases, EP encoding genes are expressed at higher levels in biofilm-forming bacteria as compared to planktonic bacteria [117]. This constitutes a serious clinical issue as EPs are key elements in the generation of multidrug resistance, often leading to chronic infections that are difficult to treat [117,118]. The involvement of EPs in biofilm formation is confirmed by the ability of EP inhibitors to abolish biofilm formation [119]. *E. coli* mutants missing the genes for several EPs, including the MFS EPs EmrD or EmrKY, exhibit low biofilm formation [120]. Similarly, *Salmonella enterica* serovar Typhimurium strains carrying mutations in *emrAB* or *mdfA* genes, both encoding MFS EPs [119] are impaired in biofilm formation. Although EPs have been considered mainly as devices used by biofilm-embedded bacteria to increase resistance to antimicrobial agents, these transporters also play an important role in the metabolism of biofilm-forming bacteria. In general, the nature of exported substrates drives biofilm formation in a positive or negative manner [26]. In fact, bacteria can use EPs to translocate substances needed to aggregate biofilm matrices and quorum-sensing molecules that affect biofilm formation. As for MFS EPs, some examples of positive impact exist (Table 1). This is exemplified by the *Helicobacter pylori* GluP transporter, which exports sugars such as d-glucose [121]. In *H. pylori* strains lacking the GluP EP the biofilm matrix contains more cavities as compared to the wild-type. As d-glucose is involved in the synthesis of bacterial exopolysaccharides, failed export of this important matrix component negatively affects the correct packaging of the biofilm [92]. Similarly, during *E. coli* biofilm growth the upregulation of *setB*, encoding an MSF EP involved in the efflux of glucose, has been surmised to support the requirement for increased export of sugars in the biogenesis of the biofilm matrix and the extrusion of non-metabolizable sugars that could be toxic to biofilm cells [89,117]. A further example in *E. coli* is represented by EmrD [91], an EP involved in the efflux of arabinose, which promotes cell aggregation an biofilm formation Another case in *E. coli* is the MDR EP TetA(C) which stimulates the production of colanic acid, a capsular polysaccharide component of the biofilm matrix favoring biofilm maturation [90].

In *S. aureus* the *proP* gene, encoding an MFS proline/betaine transporter, is upregulated in biofilm as compared to planktonic growth [93]. An increased level of ProP during the initial stage of biofilm formation may facilitate the transport of proline and betaine across the membrane protecting the cells from osmotic stress since these osmolytes are critical for bacterial cell survival under these conditions [122]. Another *S. aureus* EP involved in ensuring appropriate fitness of bacteria in biofilm embedded cells is NorB which, as already mentioned, contributes to bacterial survival in abscesses. Expression of the *norB* gene is increased in response to hypoxic conditions and low pH within biofilms [56]. In particular, it has been proposed that NorB may ensure that biofilm cells are protected from the toxic effects of organic acids produced by the fermentation of glucose during anaerobic respiration in biofilm growth.

Other MFS EPs involved in biofilm formation are found in *Acinetobacter baumannii*, a multidrug-resistant human pathogen responsible for severe nosocomial infections. The clinical relevance of *A. baumannii* is largely due to its ability to survive under stressful conditions by forming biofilms [88]. Three *A. baumannii* MFS EPs are involved in biofilm formation: Pmt, AbaF, and AbaQ. The Pmt transporter is encoded by a gene expressed at significantly higher levels in biofilm cells as compared to planktonic cells [88]. By monitoring the amount of extracellular DNA (eDNA) released by strains overexpressing *pmt*, Sahu et al. [123] proposed the involvement of the Pmt EP in nucleic acid transport. Since DNA and RNA are well-known scaffolding components of the biofilm matrix [124], the authors [123] have inferred that an increase in eDNA supports a more abundant development of the bacterial biofilm. Another MFS transporter involved in biofilm formation is AbaF, responsible for the efflux of fosfomycin. Disruption of the *abaF* gene limits the capacity to form biofilms, probably because of a reduced efflux of biofilm matrix compounds. Moreover, using the *Caenorhabditis elegans* model, it has been shown that worms infected with *A. baumannii abaF* defective strains survive longer than those infected with the wild-type strain, suggesting that *abaF* mutants have a reduced capacity to expel host-derived antibacterial factors [86]. Finally, it has been shown that the lack of *abaQ*, a gene encoding a MFS transporter, significantly reduces bacterial surface-associated motility and virulence in a *C. elegans* model [87]. Interestingly, loss of motility and reduced virulence was also observed in a biofilm-hyperproducing *A. baumanni* clinical strain deleted in *abaQ*. Considering the relevant role of bacterial motility in the first stage of biofilm generation, it is likely that AbaQ also contributes to *A. baumanni* virulence allowing an efficient biofilm formation. Altogether this evidence suggests that inhibiting the synthesis of EP components may constitute a novel effective approach to overcome the increasing emergence of biofilm-based infections.

## 7. Conclusions

In recent years, a considerable amount of knowledge on the mechanisms, regulation, and physiological functions of MFS EPs has been acquired in several bacterial systems. This has led to a deeper understanding of the varied biological roles of this EP family, which go beyond simple drug transport and also affect resistance to plant and mammalian defence systems, virulence, and community behaviors. In several cases, the major role is still not fully elucidated. However, evidence accumulated so far strongly suggests that extrusion of antibiotics is not the original physiological function of MDR EPs, which should rather be regarded as sophisticated machines contributing to optimize bacterial interactions with other cells and with the environment. The complexity of the regulatory systems underlying the expression of EPs is in line with the need to rapidly and coordinately activate expression of EP genes in response to a broad range of substrates and environmental signals. Overall, further investigations on the function of EPs can be anticipated to open new perspectives for a better comprehension of the physiology of bacterial cells and of the elaborate interplay of bacteria with their hosts.

## Figures and Tables

**Table 1:** Efflux pumps of the MFS family involved in interactions with plant, animal, and bacterial cells.

	Effux Pump *	Microorganism	Main Functions **	Reference
Interaction with plants	EmrAB	*Erwinia chrysanthemi*	Virulence in potato, resistance to phenolic acids	[70,72]
	EmrAB	*Sinorhizobium melitoti*	Response to plant induced luteolin	[73,77]
	RmrAB	*Rhizobium etli*	nodule formation, resistance to plant flavonoid	[74]
	SalRAB	*Rhizobium leguminosarum*	Resistance to salicylic acid	[63]
Interaction with animal cells	EmrKY	*Shigella flexneri*	Survival in macrophages	[66]
	EmrAB	*Escherichia coli*	Resistance to bile salts	[40]
	FarAB	*Neisseria gonorrhoeae*	Resistance to long-chained fatty acid	[45]
	MdrT	*Listeria monocytogenes*	Export of cholic acid and c-di-AMP	[51,79]
	MdrM	*L. monocytogenes*	Export of c-di-AMP	[79]
	Tet38	*Staphylococcus aureus*	Internalization and survival in epithelial cells	[53,54,80]
	NorB	*S. aureus*	Survival in abscesses and in biofilms	[56,57]
	NorD	*S. aureus*	Survival in abscesses	[58]
	P55	*Mycobacterium tuberculosi* *Mycobacterium bovis*	Survival in macrophages and in mouse model	[81,82]
	Tap	*M. tuberculosis*	Survival in macrophages	[83,84,85]
	VceCAB	*Vibrio cholerae*	Resistance to bile salts	[42]
Bacterial communication and Biofilm formation	EmrCAB	*Chromobacterium violaceum*	Quorum-sensing signaling	[37]
	AbaF	*Acinetobacter baumannii*	Biofilm formation, virulence	[86]
	AbaQ	*A. baumannii*	Surface—associated motility, virulence	[87]
	Pmt	*A. baumannii*	Nucleic acid transporter—biofilm formation	[88]
	SetB	*E. coli*	Biofilm formation	[89]
	TetAC	*E. coli*	Biofilm maturation	[90]
	EmrD	*E. coli*	Biofilm formation	[91]
	GluP	*Helicobacter pylori*	Biofilm formation	[92]
	ProP	*S. aureus*	Protection from osmotic stress	[93]
	EmrCABsm	*Stenotrophomonas maltophilia*	Extrusion of environmental molecules	[36]

* In the case of tripartite EPs, the outer membrane component is indicated only when co-transcribed in the same operon. ** With the exception of FarAB, Pmt, SetB, and ProP all EPs confer resistance to one or more antibiotics.
